# An Evaluation of Entropy Measures for Microphone Identification

**DOI:** 10.3390/e22111235

**Published:** 2020-10-30

**Authors:** Gianmarco Baldini, Irene Amerini

**Affiliations:** 1 European Commission, Joint Research Centre, 21027 Ispra, Italy; 2 Department of Computer Science, Sapienza University of Rome, 00185 Roma, Italy; amerini@diag.uniroma1.it

**Keywords:** security, identification, authentication, signal processing

## Abstract

Research findings have shown that microphones can be uniquely identified by audio recordings since physical features of the microphone components leave repeatable and distinguishable traces on the audio stream. This property can be exploited in security applications to perform the identification of a mobile phone through the built-in microphone. The problem is to determine an accurate but also efficient representation of the physical characteristics, which is not known a priori. Usually there is a trade-off between the identification accuracy and the time requested to perform the classification. Various approaches have been used in literature to deal with it, ranging from the application of handcrafted statistical features to the recent application of deep learning techniques. This paper evaluates the application of different entropy measures (Shannon Entropy, Permutation Entropy, Dispersion Entropy, Approximate Entropy, Sample Entropy, and Fuzzy Entropy) and their suitability for microphone classification. The analysis is validated against an experimental dataset of built-in microphones of 34 mobile phones, stimulated by three different audio signals. The findings show that selected entropy measures can provide a very high identification accuracy in comparison to other statistical features and that they can be robust against the presence of noise. This paper performs an extensive analysis based on filter features selection methods to identify the most discriminating entropy measures and the related hyper-parameters (e.g., embedding dimension). Results on the trade-off between accuracy and classification time are also presented.

## 1. Introduction

This paper deals with the problem of microphone identification using physical features. Identification is the process of using claimed or observed attributes to single out one entity among others in a set of identities [1]. The conventional approach in Information and Communication Technologies (ICT) to implement identification is to use cryptographic algorithms, where the identity of an electronic device is verified using a cryptographic key stored in the device itself. Even if this approach is predominantly used in modern ICT systems, it has some weaknesses, which are well known in literature [2]. Keys can be stolen or tampered with and they must be protected. Complex key management processes and systems must also be implemented to distribute the cryptographic material (e.g., private and public keys) and support their renewal in case of obsolescence or migration from one cryptographic system to another. In some cases, these requirements cannot be implemented in a cost-effective way, and the research community has proposed new approaches, including the one proposed in this paper, which is based on physical features.

This paper focuses on the identification, which is based on the intrinsic properties of the electronic device which cannot be easily stolen or cloned by an attacker. The process is based on the concept that small differences in the production of the electronic devices generate unique traces in the generated digital output. These small differences are present because of the materials used to produce the electronic components of the device (e.g., filter, amplifiers) or due to the manufacturing process. Because electronic devices are submitted to testing and quality controls before market deployment, these small changes are generally within the tolerances of the requested technical requirements (e.g., a microphone must be able to record the voice by an human being or a communication device must be able to support communication in a specific wireless standard) and can be also exploited to uniquely identify the electronic device. Findings from numerous research groups in the last decade have proven that a large variety of consumer electronic devices, like cameras, radio frequency transmission devices, sensors, and mobile phones, can be uniquely identified using the approach described above [3,4]. This approach has been called with various terms depending on the type of electronic device or the specific technique used: radiometric identification [5], Radio Frequency (RF)-DNA in Reference [6] (because the physical features resemble the DNA of an human being), fingerprints (akin to the biometric fingerprints of an human being), Sensor Pattern Noise (SPN) in cameras [7], and so on.

The focus of this paper is on the identification of a specific class of electronic devices: the microphones and more specifically the built-in microphones of mobile phones. The concept is to exploit the intrinsic features of the microphones to perform the identification of the mobile phone by stimulating the mobile phone with a specific sound and then by comparing the recordings of each microphone using the extracted features. A significant challenge for the practical application of this technique is that the validation of the identity must be implemented in a reasonable time. A sound recording can be of a relatively large size (e.g., if recorded at high frequency of 44,100 Hz in Pulse Code Modulation (PCM) mode), and the extraction of the fingerprints from such large files can be time consuming. A common approach is to implement a dimensionality reduction by extracting specific features like statistical features which reduce the classification space. For example, in Reference [6], the authors used a combined time-frequency and statistical features approach where the digital signals generated by a wireless electronic devices are transformed in the time-frequency domain with a Gabor transform and then variance, kurtosis, and Shannon entropy are extracted from segments in the time-frequency domain. On the other side, the application of deep learning to device identification has been recently applied with successful results but, on the downside, it requires a large amount of time for training. Dimensionality reduction with the extraction of specific features can be more time efficient, even if it requires the identification of adequate features for classification.

*Our Contribution*: This paper provides an analysis on the suitability of entropy measures as features for the identification of built-in microphones. The analysis is performed on an experimental dataset of 34 microphones stimulated by three different types of audio recordings, which have been created by the authors themselves. Thus, it refers to a supervised learning on a closed set. The objective is to identify the most discriminating entropy-based features and the impact of the hyperparameters in the entropy definition (e.g., embedding dimension in permutation entropy), as well as to evaluate the classification time. Results obtained from different machine learning algorithms are also compared. So far, an extensive study on entropy measures for microphone identification has not performed in literature, to the knowledge of the authors, as described in the related work Section 2. The results show that the application of selected entropy measures is able to achieve a very high identification accuracy (higher than 98% in most cases), the approach is quite robust to the presence of noise and it provides the benefit of an high dimensionality reduction, which makes it suitable to practical applications. The study presented in this paper is more focused on the identification problem (identify a microphone among a set of microphones) rather than the mitigation of a masquerading attack where a microphone must be distinguished from another microphone because the identification problem is already quite extensive.

*Structure of the paper*: The paper is composed by the following sections. Section 2 provides an overview of the related work. Section 3 describes the overall methodology, the materials used, the feature selection algorithms, the machine learning algorithms, and the related classification metrics. Section 4 describes in length all the entropy measures, which have been used for the analysis. Section 5 presents the results, where the performance of the different entropy measures is given. Finally, Section 6 provides the conclusions to this paper.

## 2. Related Work

As described in the introduction, a study on the application of entropy measures for microphone identification is lacking in literature and there are no similar studies. Then, this related work section focuses on two different areas: (a) the concept of microphone identification through its physical features and (b) the application of entropy measures for identification problems, as in this paper, to other domains.

### 2.1. Microphone Identification Using Physical Features

Identification of microphones of mobile phones on the basis of voice sound recordings has been investigated in the research community for forensics purposes, as well as for identification/authentication. In the forensics case, the problem is to identify the source microphone (or at least the model) on the basis of an available sound recording, which has been uploaded to the web or found on a crime scene (e.g., a computer in the house of the potential criminal). The sound recording can also be extracted from a video. The pioneering work in this field is Reference [8], wherein the authors proposed a set of audio steganalysis-based features to cluster (K-means) or to predict (Naive Bayes classifiers) both the microphone and the environment. The work has been extended in Reference [9], wherein a first proof of concept concerning the usage of information fusion in microphone classification has been proposed. The proof of concept was based on the combination of statistical features (by means of supervised classification) and unweighted information fusion (at match, rank, and/or decision level). A common approach for microphone identification is to use the spectral domain for microphone identification. For example, in Reference [10], the classification algorithm is applied to the Fourier transform of the digitized audio recordings. In a similar way, the linear- and mel-scaled cepstral coefficients are used in Reference [11] where the approach results in an high identification accuracy. The reason why the spectral domain is used in the papers referenced above, is related to the consideration that the fingerprints of the microphones are mostly dependent to the frequency responses of the internal components of the microphone, like filters and amplifiers. For the same reason, the analysis presented in this paper is based on the application of entropy measures on the spectral domain representation.

The papers above were mostly focused on forensics analysis, but the same fingerprints or discriminating features can also be used for identification as an alternative or to complement cryptographic algorithms. In particular, identification with physical features is quite similar to the Physical Unclonable Functions (PUF) concept, which can be based on the random start-up value of an uninitialized static Static Random Access Memory (SRAM) or the small variations of a Ring Oscillator (RO). In the PUF concept, the fingerprints are often inserted intentionally in the electronic device to give a greater control on the challenge-responses space [12,13]. The concept of PUF has been explored significantly in recent times. In particular, a recent paper, Reference [14], investigated different transforms for reliable secret-key binding in ring oscillator PUFs by using the fuzzy commitment scheme. The study presented in this paper does not attempt to investigate the secret-key binding or generation scheme from the physical fingerprints of the microphones. It also does not aim to address the authentication problem to mitigate an adversary attack where a microphone is manipulated or fabricated to replicate another microphone. This paper specifically aims to analyze the suitability of entropy measures to identify microphones where the fingerprints were not embedded in the design phase, but they are spontaneously generated in the design/manufacturing process.

Two recent papers have explored the exploitation of fingerprints for identification purpose. In Reference [15], the authors propose a lightweight device identification protocol (named speaker-to-microphone (S2M)) by leveraging the frequency response of a speaker and a microphone from two wireless IoT devices as the acoustic hardware fingerprint. In Reference [15], the entire frequency domain of the audio recordings is used for identification and authentication, which can be quite time consuming. This is the reason why the focus of this paper is to improve the time efficiency of the identification process by using statistical features based on entropy.

While in forensics analysis, voice audio recordings are used, in the identification contexts, any sound stimulus can be used. The authors in Reference [16] use non-voice recordings and apply spectrograms to sample recordings to characterize the recording device. The authors use a controlled set of microphones in mobile phones, as in this paper (31 phones in Reference [16] versus 34 in this paper). The authors in Reference [16] do not apply entropy or other dimensionality reduction techniques. While the obtained classification accuracy is quite high (near 100% in ideal situations), the computing time is much higher than applying dimensionality reduction using entropy as in this paper (see Section 5), where a similar degree of accuracy is anyway obtained. In a similar way, the authors of Reference [17] used the constant Q transform (CQT) to voice recordings rather than the spectrogram and then applied Convolutional Neural Networks (CNN) for classification obtaining an high identification accuracy and robustness of noise. In comparison to this paper, the application of CQT is significantly slower than Fast Fourier Transform (FFT). In addition, this paper uses non-voice recordings instead of the voice recording of Reference [17].

Sound stimuli applied to microphones for identification and authentication have also been used in Reference [18], where a Deep Learning approach based on CNN is adopted. Tones at 1 KHz and 2 KHz are used as stimuli of a set of 32 mobile phones. The results show that CNN applied to the frequency domain representation of the audio recordings are able to outperform significantly the time domain representation and shallow machine learning algorithms even in presence of noise. On the other side, the application of deep learning techniques requires substantial time consumption and computing resources for training and classification. For this reason, it is not easily applicable to the smartphone identification task when thought about in a real scenario perspective.

As shown in the identified references above, microphone recognition is often based on the application of the Fourier transform domain of the audio recordings. In some references, the spectrogram [16] or other time frequency transforms (e.g., CQT in Reference [17]) is also used, which has a justification on the consideration that audio signals are usually nonstationary. On the other side, the application of spectrogram would enlarge the space on which the entropy measures must be calculated, which would increase the analysis and classification time. In addition, the spectrogram or other time-frequency transforms have hyperparameters (e.g., window size, window type) which should be tuned, which makes the analysis even more complex. For these reasons, the authors have postponed the application of spectrogram or other time-frequency transform to future developments (see Section 6).

### 2.2. On the Application of Entropy Measures for Identification Problems

This subsection reviews the literature of the entropy measures used in this paper (e.g., Sample Entropy, Approximate Entropy, Permutation Entropy, Dispersion Entropy, and Fuzzy Entropy) for the purpose of detection and identification in different domains. The concepts of the different entropies are only briefly introduced in this section as they are described in detail in Section 4. Permutation Entropy (PeEn) was initially introduced by Bandt and Pompe in Reference [19], and it has been used for many different applications since then. In Reference [20], it was used for the identification of multiple faults in gearbox. Regarding device identification, PeEn was used for emitter identification (e.g., wireless communication system identification) in Reference [21], and, in a similar way, it has been used for radio frequency fingerprinting of wireless communication systems in Reference [22]. These two papers have a similar approach to this paper as they exploit the physical features of electronic devices for identification, and they use PeEn as a discriminating feature.

Sample Entropy (SaEn) and Approximate Entropy (ApEn) has been extensively used in the analysis of physiological signals, where it has often demonstrated superior performance. Even if there are many papers in literature using SaEn and ApEn, we select the two following works since they are similar to our approach as they compare the discriminating power of different entropy measures for classification purposes. The authors in Reference [23] used approximate entropy with other entropy measures for the identification of focal electroencephalogram signals. The entropy measures have been applied to the intrinsic mode functions generated by the application of empirical mode decomposition, while, in this paper, the Fourier Transform is used. As in this paper, Support Vector Machine (SVM), together with other machine learning algorithms, is used for classification. SaEn and other entropy measures have also been used in automatic sleep classification [24]. Contrary to this paper, which is focused on supervised classification, the authors of Reference [24] applied entropy measures for unsupervised classification.

Dispersion Entropy (DiEn) has been recently introduced by the authors in Reference [25], and it is suggested as an improvement both to PeEn and SaEn. Since its introduction, it has been applied to identification problems in different domains. In particular, for the identification and authentication of wireless communication devices, DiEn has demonstrated an improvement in the classification performance and robustness in presence of noise [26]. DiEn has also been used to detect and identify gear faults in mechanical related applications, where it has shown its superior performance in comparison to PeEn and ApEn with the additional advantage of a faster computational time [27].

In the field of audio recognition (which is related but it is not exactly the same topic of this paper), Fuzzy Entropy (FzEn) was used to identify speech against a noisy background in Reference [28]. It is shown that FzEn is particularly robust against the presence of noise, which is a reason why it was also used in this paper.

## 3. Materials and Methods

### 3.1. Materials

The set of materials used in this paper is composed by audio recordings generated by the built-in microphones of 34 mobile phones belonging to different brands and models. All the models have only one built-in microphone. While the set of mobile phones is quite extensive for different types and models, it also contains a large set of mobile phones of the same model (i.e., Samsung ACE) to test the most challenging case of intra-model microphone classification rather than inter-model classification. Table 1 describe all the mobile phones used in the experiment.

Each of the 34 mobile phones was stimulated by three separate audio signals (e.g., sounds) to evaluate the classification accuracy: (a) gunshot (recorded shot by a gun), (b) the sound produced by a pneumatic hammer, and (c) a tone at 1 KHz registered for two seconds, increasing in amplitude for one second and then decreasing for another second.

In the rest of this paper, the dataset generated with the tone at 1 KHz is called 1 K, the data set generated with the gunshot audio stimulus is called GUNSHOT and the dataset generated with the pneumatic hammer audio stimulus is called PNE.

These sounds were chosen because of the extensive frequency extension and from the fact that they are well suited to stimulate a wide microphone frequency response with respect to a pure only-voice stimulus. In addition, they are quite different among themselves, as the gunshot is similar to a pulse, the pneumatic hammer has the characteristics of a repeated pulses, and the tone is a sinusoidal signal.

**Figure 1:**
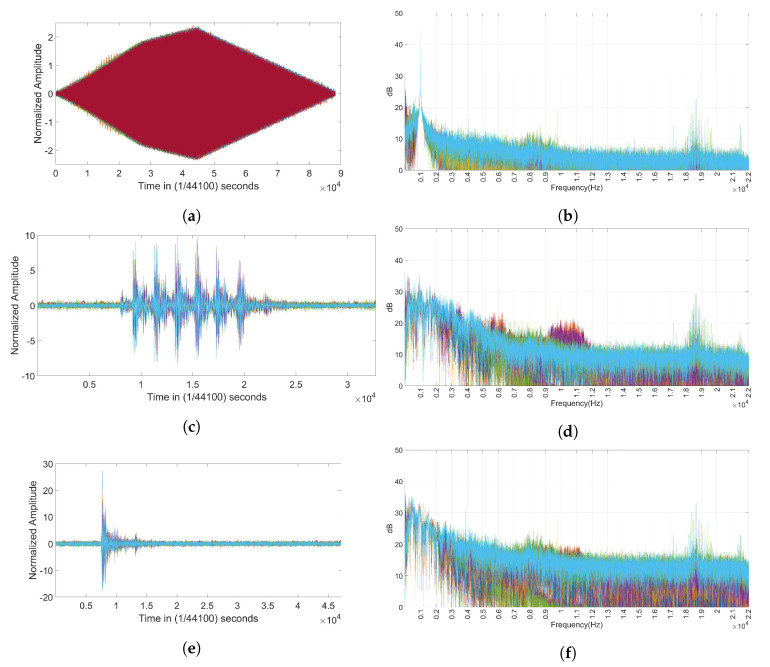
Audio stimuli representations in the time domain and correspondent spectral representation. (**a**) Tone at 1 KHz audio recordings for the 34 microphones: Time domain; (**b**) Tone at 1 KHz audio recordings for the 34 microphones: Frequency domain (complex magnitude); (**c**) Pneumatic hammer audio recordings for the 34 microphones: Time domain; (**d**) Pneumatic hammer audio recordings for the 34 microphones: Frequency domain (complex magnitude); (**e**) Gunshot audio recordings for the 34 microphones: Time domain; (**f**) Gunshot audio recordings for the 34 microphones: Frequency domain (complex magnitude).

The time domain representation of the signals recorded for all the considered mobile phones is shown in Figure 1a,c,e, respectively, for the 1 K, PNE, and GUNSHOT datasets. The frequency (complex magnitude) representation of the signals recorded for all the considered mobile phones is shown in Figure 1b,d,f, respectively, for the 1 K, PNE, and GUNSHOT datasets. We note that the spectral representations of the PNE and GUNSHOT datasets (Figure 1d,f) is relatively similar (even if some differences are present around 6 KHz and 10 KHz). Time-frequency analysis can highlight more significant differences but this would require longer processing times as mentioned before and it is reserved for future developments (also see Section 6).

**Figure 2:**
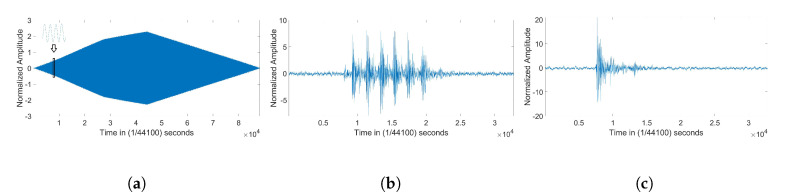
Time domain representation taken from phone 22 for the the tone at 1 KHz (1 K), pneumatic hammer audio stimulus (PNE), and gunshot audio stimulus (GUNSHOT) datasets. (**a**) 1 K; (**b**) PNE; (**c**) GUNSHOT.

Since the combination of all the 34 phones may be difficult to distinguish, Figure 2 shows a single plot (taken from mobile phone 22) for each of three datasets.

Each audio signal is repeated 800 times with a 1-s separation among them. This approach is similar (but with different sound stimuli) to what was used in Reference [15,16]. Each mobile phone was configured to be at the same distance from the amplifier. The recording frequency of the phone was set to 44,100 Hz and the recordings were saved in PCM format. Regarding the 1 K dataset, only the decimation at $D_{R}=20$ was used because the stimulus is composed only by a tone at 1 KHz and higher values of decimation ($D_{R}=40$ and $D_{R}=60$) would have filtered the main frequency response.

### 3.2. Overall Methodology

**Figure 3:**
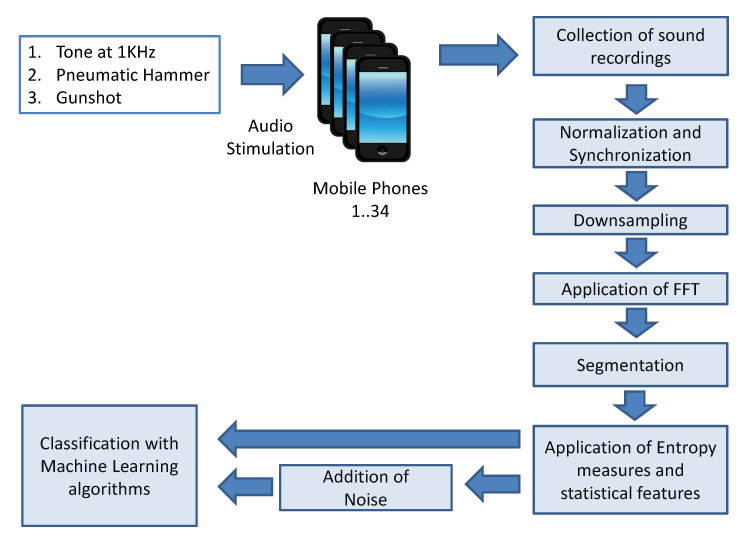
Overall methodology for microphone identification.

The overall methodology used to collect the audio recordings, process them to extract the fingerprints and to compare the classification results is described in the following paragraphs and is pictured in Figure 3.

In the first step, each of the 34 mobile phones was stimulated by three different types of audio signals. As described before in Section 3.1, the three sounds used in the analysis are based on the sounds generated by (a) gunshot, (b) the sound produced by a pneumatic hammer, and (c) a tone at 1 KHz, increasing in amplitude and then decreasing in amplitude in two seconds.

The audio recordings from each mobile phone were synchronized (the first phone was used as a reference) and normalized. Pearson correlation was used to synchronize the recordings.

The discrete Fourier transform (DFT) was applied to the recordings using a Fast Fourier Transform (FFT) algorithm. As described in the Section 2 the classification usually exploits the frequency response because it captures the discriminating features of the microphones (due to filters and to the physical shape of the microphones) in a better way than the original time domain representation [16,18].

The initial sample rate in PCM format is set to 44,100 Hz, which would generate very large files and a long classification time if the FFT is applied directly to the digital representation of the audio recordings. Then, the audio recordings were first downsampled through decimation, and then the FFT was applied. Three different values of decimation were used in the analysis conducted in this paper: $D_{R}=20$, $D_{R}=40$, $D_{R}=60$. Because the classification is performed on the output of the FFT rather than to the original time representation of the audio recording, the application of decimation is used for dimensionality reduction: to decrease the time needed to calculate the entropy measures and to make more efficient the classification. Decimation may introduce aliasing to reconstruct the original signal according to the Nyquist-Shannon theorem if the sampling rate is twice the highest frequency of the original analog signal (i.e., the audio recording). On the other side, the problem to solve in this study is not to specifically recover the original analog signal from the frequency domain representation but to classify the microphones on the basis of their frequency response and entropy measures.

As shown in the results presented in this paper (see Section 5), there is a trade-off between classification and decimation because higher decimation values may correspond to a lower classification performance. This is an obvious consequence of the consideration that the decimation filters out the high frequency components of the signal, which may contains the “fingerprints” of the microphone needed to discriminate a microphone from another. On the other side, it is not known a priori where the fingerprints are located in the spectral domain. For these reasons, one of the objectives of this paper is to evaluate the impact of decimation on the classification performance.

The results of the analysis have shown that the complex magnitude spectral component of the Fast Fourier Transform (FFT) has significant more discriminating power than the phase component. Then, the statistical and entropy features are applied only to the complex magnitude component. This result is consistent with the findings in literature on microphone identification [11,16,18], where the complex magnitude components of the spectral domain are used. The reason for this behavior is linked to the frequency response of the hardware elements of the microphones (e.g., filters, amplifiers), in which “fingerprints” are more relevant in the complex magnitude component rather than the complex phase component of the spectral representation.

Different types of entropy measures were applied to the sound recordings. The details on the types of entropy measures and to the related hyperparameters are described in detail in Section 4. In addition, standard deviation, skewness, and kurtosis were also calculated. These statistical features were used since they are often employed in electronic components classification based on the fingerprinting concept [29,30]. Standard deviation, skewness, and kurtosis are used as baseline benchmark for the entropy measures. The results in Section 5 show that the entropy measures improve significantly the classification performance in comparison to standard deviation, skewness, and kurtosis.

We highlight that the microphone identification approach is heuristic and not based on a theoretical model as this would be too complex to represent all the different components (i.e., filter, amplifiers, diaphragm, magnet) of the microphone and their interconnections. The microphone fingerprints are not known a priori, but they are related to the different frequency responses of the microphone, which are based in turn to the small physical differences in the components of the microphone and how they are connected. The entropy measures capture the small differences in the frequency response of the different microphones. It is demonstrated in this paper (see Section 5) that they have significant discriminant power to provide high identification accuracy.

Three different machine learning algorithms were used for classification: SVM, K-Nearest Neighbor, and Decision Tree. Details on the application of the machine learning algorithms are provided in Section 3.4.

One important parameter for the evaluation of the approach proposed in this paper is the robustness of the microphone identification in presence of noise because, in practical conditions, the audio signal which stimulates the microphone will be received in presence of background noise of different types. It is almost impossible during the recording phase to reproduce all the possible real types of noise or to simulate attenuation between the audio source and the microphone. Thus, the most common approach used in literature is to add noise to the recorded audio samples to simulate the presence of noise during the recording phase. This is the approach used in Reference [16,18,31], where a similar argument is proposed. Additive White Gaussian Noise (AWGN) was added to the audio recordings with decreasing values of Signal Noise Ratio (SNR) to simulate the presence of attenuation due to path loss and background noise. In addition, we point out that the audio recordings composing the datasets proposed in this paper are already recorded in non-ideal conditions where background noise (people talking in the laboratory or noise from vehicles passing close to the laboratory) was already present.

### 3.3. Feature Selection Algorithms

Two main feature selection algorithms are used in this study: ReliefF [32] and Neighborhood Component Analysis (NCA) [33]. Both algorithms are filter selection methods where the selection of the features is independent of the machine learning algorithms subsequently used for classification.

ReliefF finds the weights of predictor features in a multi-class categorical dataset. The ReliefF algorithm is a peculiar filter algorithm, which finds the weights of predictor features in a multi-class categorical dataset. The algorithm penalizes the features that give different values to neighbors of the same class, and rewards features that give different values to neighbors of different classes. Details on the ReliefF algorithm are provided in Reference [32]. The main concept is that ReliefF first sets all feature weights to 0 and considering a dataset $X=x_{1},x_{2},...,x_{N}$ the algorithm iteratively selects a random observation $x_{r}$ from the dataset, finds the K-nearest observations to $x_{r}$ for each class, and updates, for each nearest neighbor $x_{q}$, all the weights for the predictor features. In this study, the value of K has been set to 1.

NCA is a non-parametric and embedded method for selecting features where the goal is to maximize prediction accuracy of regression and classification algorithms [33]. In this paper, NCA is used for classification. NCA aims at learning a distance metric by finding a linear transformation of input data such that the average leave-one-out (LOO) classification performance is maximized in the transformed space. In classification, NCA tries to find a feature weighting vector that maximizes the average leave-one-out cross-validation accuracy. As described in the methodology Section 3.2, the feature space is based on the application of entropy measures for each segment in the spectral domain. Then, the output of the analysis presented in the results Section 5 will be an histogram of the optimal features identified for all the combined segments.

### 3.4. Machine Learning Algorithms and Classification Metrics

The following machine learning algorithms have been used to evaluate the performance of the identification and authentication process.

- SVM is a supervised learning model with the related learning algorithms that classify data by creating a hyperplane or set of hyperplanes in a high- or infinite-dimensional space, to distinguish the samples belonging to different classes. Since this paper addresses a multi-class machine learning problem, a multi-class SVM is used, which is based on an Error-Correcting Output Codes (ECOC) classifier for multi-class learning, where the classifier consists of multiple binary learners. In particular, we used the OneVsOne approach where, for each binary learner, one class is positive, another is negative, and the algorithm ignores the rest. Various kernels were tried, and the one providing the best performance was the Radial Basis Function (RBF) kernel, where the values of the scaling factor γ must be optimized together with the parameter C [34].
- K Nearest Neighbor (KNN) is an approach to data classification that estimates how likely a data point is to be a member of one class or another depending on what group the data points nearest to it are in. The KNN is an example of a lazy learner algorithm, meaning that it does not build a model using the training set until a query of the dataset is performed. The main hyperparameter in KNN is the K factor, which must be optimized for the specific classification problem. The type of distance metric used to calculate the ’nearest’ must also be chosen carefully.
- Decision tree is a predictive modeling approach where a decision tree (as a predictive model) analyze the observations about an item (represented in the branches) to reach conclusions about the item’s target value (represented in the leaves). In this case, we use classification trees where leaves represent class labels and branches represent conjunctions of features that lead to those class labels. The hyperparameter chosen for optimization is the maximum number of branches at each split. It was also chosen the option that the algorithm trains the classification tree learners without pruning them.

To minimize over-fitting and improve generalization, a 10-fold approach is used for all the machine learning algorithms where the initial data is shuffled randomly and it is split in 10 groups. For each unique group, 1/10 of the dataset is used as a test data, and the remaining 9/10 of the dataset is used as a training data. A model is created on the training set and evaluated on the test set. The evaluation score is maintained and the model is discarded. The process is repeated 10 times with the rule that each observation in the data sample is assigned to an individual group and stays in that group for the duration of the procedure (i.e., each sample is given the opportunity to be used in the hold out set 1 time and used to train the model 9 times). The evaluation scores are averaged to produce the final evaluation score.

The performance of each of the machine learning algorithms is based on the proper choice of the hyperparameters. The optimal values were identified using a grid approach across the folds and the maximum occurrence of the hyperparameters values in the resulting histogram. The chosen hyperparameter values are provided in Table 2, where the optimal values of the hyperparameters are shown together with the range over which they were calculated. All features were used for this analysis.

Finally, regarding the metric used for identification purpose, the accuracy is used, which is defined as:

(1) $$Accuracy=\frac{TP+TN}{\left(TP+FP+FN+TN\right)},$$

where TP is the number of True Positives, TN is the number of True Negatives, FP is the number of False Positives, and FN is the number of False Negatives. To complete the accuracy metric, confusion matrices are also provided to assess the predicted values against the true values. In the confusion matrices presented in this paper, each row of the matrix represents the instances in a true class, while each column represents the instances in an predicted class.

## 4. Entropy Measures

This section describes the entropy measures, which have been adopted for the analysis presented in this paper. In addition to the entropy measures, other statistical features, usually adopted for device identification, are used, including standard deviation, skewness, and kurtosis [6]. The entropy measures identified and described here are a subset of a larger set of entropy metrics, which have been empirically evaluated. In particular, Tsallis entropy was evaluated, but it did not provide a good classification performance and was discarded in the analysis presented in this paper. In addition, as the goal of the study is dimensionality reduction, it was purposely decided not to conduct an analysis by using multi-scale entropy. This decision is based on the consideration that the initial data samples are already down-sampled, which would limit the applicability of multi-scale entropy measures as the resulting length of the spectral domain representation would not be long enough.

In the following subsections, the definitions of the entropy measures are provided. For all definitions, the original spectral representation (complex magnitude) of the audio recording is defined by the series $x=x_{1},x_{2},...,x_{N}$ of length N.

### 4.1. Shannon Entropy

The definition of Shannon Entropy is provided in Equation (2).

(2) $$ShEn=-\sum_{i}^{N}p\left(x_{i}\right)log\left(p\left(x_{i}\right)\right),$$

where $p(x_{i})$ is the probability $p(x=x_{i})$.

The MATLAB implementation of the entropy from Mathworks was used.

### 4.2. Renyi Entropy

The definition of Renyi Entropy is provided in Equation (3).

(3) $$ReEn=\frac{1}{1-\alpha }log\left(\sum_{i}^{N}p{\left(x_{i}\right)}^{\alpha }\right),$$

where $p(x_{i})$ is the probability $p(x=x_{i})$. The limit for α⟶1 is the Shannon Entropy defined above. In this paper, we adopt the values of α=2,3,4 as these are the range of values used in literature [35].

The MATLAB implementation of the Renyi entropy available in Reference [36] was used.

### 4.3. Permutation Entropy

PeEn was introduced by Bandt and Pompe in their seminal paper [19]. The concept is to define an entropy measure, which takes into account the time causality of the time series (causal coarse grained methodology) by comparing neighboring values in a time series. Then, PeEn is the Shannon entropy of a sequence of ordinal patterns—the latter being discrete symbols that encode how consecutive time series entries relate to one another in terms of position and value, and it is defined by the following equation:

(4) $$PeEn=-\sum_{i}^{N!}p_{i}^{{}^{\prime }}log\left(p_{i}^{i}\right),$$

where $p_{i}^{{}^{\prime }}$ represents the relative frequencies of the possible patterns of symbol sequences, termed permutations. The permutation is related to a sequence of *m* (embedding dimension) values of the original series. A time delay τ can be used in the generation of the permutations from the original series, but, for simplicity, we set the value of τ=1 in this paper, while the value of *m* is an hyperparameter to be optimized. Additional details on the definition of the PeEn are provided in Reference [19].

The MATLAB implementation of the Permutation entropy provided by the authors in Reference [37] was used in this paper.

### 4.4. Dispersion Entropy

DiEn was recently introduced in Reference [27], and it addresses the potential weakness of PeEn where the mean value of amplitudes and differences between amplitude values are not considered in its definition. In dispersion entropy, the initial series $X=x_{i},x_{i+1},...,x_{N}$ is mapped to *c* classes. While this mapping can be implemented with various linear or non-linear approaches, the authors in Reference [27] propose to use Normal Cumulative Distribution Function (NCDF) to map x to the *c* classes. Then, the implementation of the DiEN is similar to PeEn, with the generation of dispersion patterns rather than permutations and with the calculation of the probabilities $p(\pi_{j})$ on the basis of an embedding dimension *m* and the time delay τ. As in the case of PeEn, we set τ=1 for simplicity. It is important to note that the number of possible dispersion patterns that can be assigned to each time series is set to $c^{m}$ as this links two main hyperparameters in the application of DiEn and creates a constraint on such values because $c^{m}$<N. Then, the Shannon Entropy (ShEn) is applied to the probabilities of the dispersion patterns in a similar way to the implementation of PeEn where permutations are used:

(5) $$DiEn=-\sum_{j}^{c^{m}}p\left(\pi_{j}\right)log\left(p\left(\pi_{j}\right)\right).$$

The MATLAB implementation of the Dispersion entropy provided by the authors in Reference [25,37] was used in this paper.

### 4.5. Approximate Entropy

ApEn was initially proposed by Pincus in Reference [38] and it is related to the predictability or regularity of a time series. It was devised as an approximation of the Kolmogorov entropy of an underlying process. The algorithm to define ApEn is a search for the repetitive patterns of length *m* commencing at sample i in which the distance induced by the maximum norm differs up to an error threshold *r*. Then, ApEn is defined by the following equation:

(6) $$ApEn\left(m,r,N\right)=\Phi^{m}\left(r\right)-\Phi^{m+1}\left(r\right),$$

where:

(7) $$\Phi^{m}\left(r\right)=\frac{1}{\left(N-m+1\right)}\sum_{i=1}^{N-m+1}log\left(C_{i}^{m}\left(r\right)\right),$$

with ${C_{i}}^{m}\left(r\right)$ is the number of vectors $x_{i}\in {ℜ}^{m}$ such that the distance $d(x_{i},x_{j})<r$ and $x_{i}=x_{i},x_{i+1},...,x_{i+m-1}$;

The MATLAB implementation of the Approximate entropy provided by the authors in Reference [39,40] was used in this paper.

### 4.6. Sample Entropy

SaEn was defined as an evolution of ApEn with the objective to solve the bias of ApEn due to counting self-matches, and it was shown to exhibit better statistical properties than ApEn in many cases [41]. It is computed in a similar fashion than ApEn described in the previous Section 4.5, but the final step of calculating SaEn becomes:

(8) $$SaEn\left(m,r,N\right)=-log\left(\frac{A^{m}\left(r\right)}{B^{m}\left(r\right)}\right),$$

where $B_{m}\left(r\right)$ is defined as the mean of the number of vectors $x_{i}\in {ℜ}^{m}$ such that the distance $d(x_{i},x_{j})<r$ with i≠j divided by (N−m+1). The value of $A_{m}\left(r\right)$ is defined in the same way with $x_{i}\in {ℜ}^{m+1}$.

The MATLAB implementation of the Sample entropy provided by the authors in Reference [41,42] was used in this paper.

### 4.7. Fuzzy Entropy

This paper also applies the FzEn defined by the authors in Reference [43], where is reported that even if SaEn is slightly faster than FzEn, the latter is more consistent and less dependent on the data length of the series where it is applied. Fuzzy Entropy is based on similar concept of SaEn and ApEn but the number of vectors which satisfy the distance condition in comparison to the tolerance *r* is calculated using a fuzzy function of this form:

(9) $$\mu =e^{{-d}^{p}/r},$$

where *p* is set to 1 in the analysis done in this paper.

Then, the *FzEn* is calculated as:

(10) $$FzEn\left(m,p,r,N\right)=-log\left(\frac{\alpha^{m}\left(r\right)}{\beta^{m}\left(r\right)}\right),$$

where α is related to ${ℜ}^{m+1}$, and β is related to ${ℜ}^{m}$.

The MATLAB implementation of the Fuzzy entropy provided by the authors in Reference [42] was used in this paper.

### 4.8. Choice of the Hyperparameters for the Entropy Measures

Each entropy measure identified above is based on the determination of the value of hyperparameters. This section discusses the choice of the value of the hyperparameters in relation to the length N of the spectral domain representation of the audio recording to be analyzed. All the entropy measures apart from ShEn are based on the embedding dimension *m*. An obvious consideration is that N>>m, in order to have a significant number of patterns to estimate the entropy. DiEn is also based on the parameter *c* (i.e., the number of classes). In Reference [25], the authors recommend that $N>c^{m}$. An increase on *m* does also increase the computing time in the application of entropy features, which goes against the principle of this paper to make the identification and authentication process time efficient. On the other side, the parameter $D_{R}$ influences the choice of the values of *c* and *m* because the length of the spectral representation (complex magnitude) is dependent on this parameter (the greater is the value of $D_{R}$ and the shorter is the length). Then, the values of *m* and *c* must be tuned accordingly to the value of $D_{R}$. In turn, this aspect influences the number of features used. To address these requirements on the hyperparameters, different combinations of such parameters were used as defined in Table 3.

**Figure 4:**
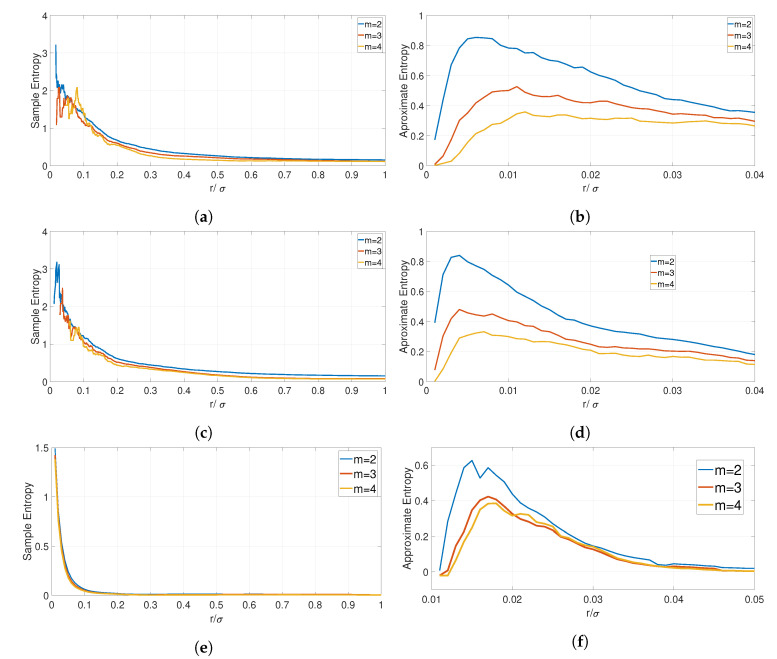
Sample and Approximate Entropy for $D_{R}=20$ on the ratio between r and the standard deviation for the PNE, GUNSHOT and 1 K datasets. (**a**) Sample Entropy diagram in relation to the r factor for the PNE dataset; (**b**) Approximate Entropy diagram in relation to the r factor for the PNE dataset; (**c**) Sample Entropy diagram in relation to the r factor for the GUNSHOT dataset; (**d**) Approximate Entropy diagram in relation to the r factor for the GUNSHOT dataset; (**e**) Sample Entropy diagram in relation to the r factor for the 1 K dataset; (**f**) Approximate Entropy diagram in relation to the r factor for the 1 K dataset.

The application of ApEn, SaEn, and FzEn is based on the tolerance, such as the threshold value *r*. In literature (see Reference [41]), it is recommended to use values of $r>0.1*std(X_{N})$ where $std(X_{N})$ is the standard deviation of the series. This value may change depending on the specific characteristics of $X_{N}$, thus in this paper, the value of *r* is determined or validated using the approach presented in Reference [44]. A similar analysis has been conducted to evaluate when the maximum values of the Approximate Entropy are reached in relation to the ratio between the *r* and the standard deviation. The results are shown in Figure 4 and, more specifically, in Figure 4a for the PNE dataset, Figure 4c for the GUNSHOT dataset, and Figure 4e for the 1 K dataset. The graphs are calculated on a random burst from the 34 phones with $D_{R}=20$ (see Section 3.2 for the definition of $D_{R}$), but similar results are obtained for other random selected bursts. The results are consistent among the figures, and they show that the maximum of the SaEn is achieved for a value of r/σ < 0.1 across the datasets, and this is the reason why r/σ>0.1 is used. A similar study was done for the Fuzzy Entropy, and it is not reproduced here for space reasons.

The study for the ApEn is also consistent across the different datasets, and it is shown in Figure 4b for the PNE dataset, Figure 4d for the GUNSHOT dataset, and Figure 4f for the 1 K dataset. The results show that the peak of the ApEn is obtained for values of r/σ < 0.03. For this reason, values of r/σ > 0.03 are used.

Then, on the basis of the previous definitions of the entropy measures, we identify, in Table 4 for $D_{R}=20$ and Table 5 for $D_{R}=40$ and $D_{R}=60$, the list of the features used in the study with the related hyperparameters. Because the application of $D_{R}=20$ has a larger number of combinations of *m* and *c* (see Table 3), the number of applied features is larger than for the application of $D_{R}=40$ and $D_{R}=60$. In particular for $D_{R}=20$, 41 features are calculated, while, for $D_{R}=40$ and $D_{R}=60$, 39 features are employed.

## 5. Results

This section provides the results for the classification of the microphones based on the entropy features identified in Section 4 in addition to selected statistical features (e.g., standard deviation, skewness, and kurtosis). This section is composed of three subsections. Section 5.1 describes the results from the application of the feature selection process described in the Section 3.3, both for the ReliefF and the NCA algorithms. Section 5.2 provides the preliminary results on the identification accuracy using all the described entropy measures for different values of noise expressed as Signal to Noise Ratio (SNR). A comparison of the classification performance of the different machine learning algorithms is provided in Section 5.3, where the optimal machine learning algorithms for each dataset are chosen. Section 5.4 complements the previous accuracy results reporting the confusion matrices for all 34 phones with respect to the different datasets.

As mentioned before, to evaluate the robustness of the algorithms against the presence of noise, the analysis is performed on the datasets where AWGN is progressively added to the initial recording to simulate decreasing values of SNR expressed in dB. While different combinations of training sets and test sets with noisy and not noisy conditions are possible, this analysis uses training and test set with the same value in dB of the SNR. One reason for this choice is because noise may impact significantly the estimation of entropy, and we wanted to compare the same kind of data in the classification process.

### 5.1. Results on Feature Selection

**Figure 5:**
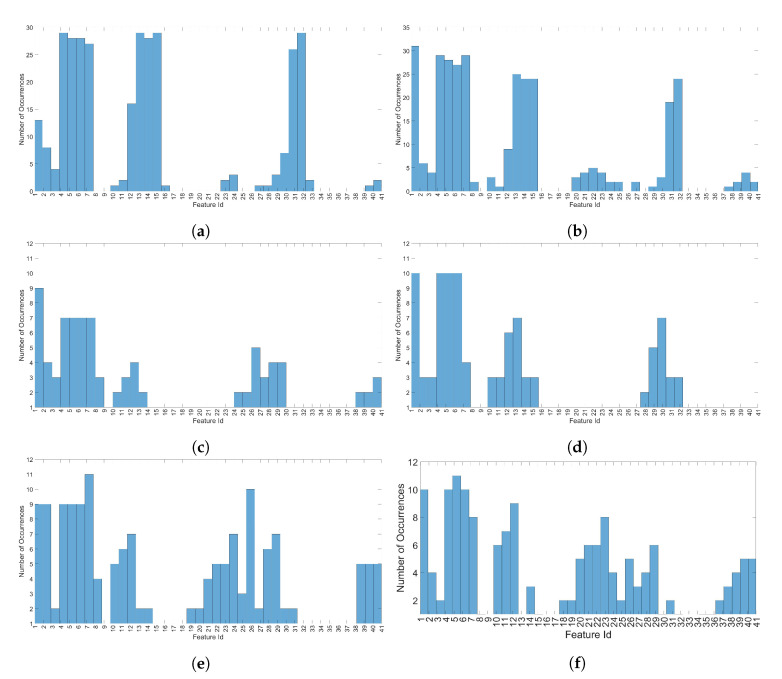
Optimal features for all datasets using ReliefF and Neighborhood Component Analysis (NCA) for $D_{R}=20$. (**a**) Histogram of the ten best (highest ranking) features set with ReliefF and $D_{R}=20$, 1 K dataset; (**b**) Histogram of the ten best (highest ranking) features set with NCA and $D_{R}=20$, 1 K dataset; (**c**) Histogram of the ten best (highest ranking) features set with ReliefF and $D_{R}=20$, PNE dataset; (**d**) Histogram on the set of the ten best (highest ranking) features with NCA and $D_{R}=20$, PNE dataset; (**e**) Histogram of the ten best (highest ranking) features set with ReliefF and $D_{R}=20$, GUNSHOT dataset; (**f**) Histogram of the ten best (highest ranking) features set with NCA and $D_{R}=20$, GUNSHOT dataset.

The analysis on the classification power of the entropy features (to identify the most discriminating ones) is one of the goals of this paper. As described in Section 3.3, two different selection features algorithms have been evaluated: ReliefF and NCA. The results on the application of these feature selection algorithms are described in detail in Figure 5 for a value of $D_{R}=20$. The other values of $D_{R}$ are not reproduced here for space reasons, but the list of the best 10 features (which were used in the classification) are anyway summarized in Table 6 for all different values of $D_{R}$. The specific charts in Figure 5 show the histograms on the occurrence of the 10 best features among all the segments in which the spectral domain representation of the signal has been represented (complex amplitude only). For example, a value of 10 for Feature Id = 6 in Figure 5d means that the standard deviation feature was present 10 times in the top ten ranked selected features from the output of the NCA algorithm. The value of 10 features was chosen because the analysis of the weights and rankings from both ReliefF and NCA algorithms has shown that additional features beyond the first 10 features in ranking did not add significant discriminant power in the classification process. Figure 5a,c,e show the results from the application of the ReliefF algorithm, respectively, for the 1 K, PNE, and GUNSHOT dataset. Figure 5b,d,f show the results from the application of the NCA algorithm, respectively, for the 1 K, PNE, and GUNSHOT dataset.

The results from both the feature selection algorithms are consistent and they identify a common set of entropy measures, which have more discriminating power. In particular, the Renyi, Shannon, and Approximate Entropy are particularly relevant for the 1 K dataset and for the GUNSHOT dataset. The Renyi, Shannon, Approximate Entropy, and Dispersion Entropy are relevant for the PNE dataset. In addition, variance and skewness do also contribute to a strong classification performance for the PNE and GUNSHOT datasets. Even if other features (Sample Entropy, Permutation Entropy, and Fuzzy entropy) do also appear in the results, their recurrences is lower. These results are obviously specific for the problem of microphone identification and for the specific dataset. On other side, three different sound stimuli were used on a relatively large set of microphones from different brands, which support a potential generalization of these results. We hope that such results could help the research community to evaluate the classification performance of these features for other datasets of microphones.

As mentioned before, the full list of the sets of optimal features from ReliefF and NCA for the 1 K, PNE, and GUNSHOT dataset are presented in Table 6.

On the basis of the results obtained above, we provide in the next subsections the results for the classification performance for microphone identification.

### 5.2. Accuracy Results

This section discusses the results of the evaluation of microphone identification for each value of $D_{R}$ ($D_{R}=20,D_{R}=40,D_{R}=60$) using all the features, the features selected as a results of the feature selection process (i.e., ReliefF and Neighborhood component analysis (NCA)) and the statistical features (variance, kurtosis, and skewness). For the 1 K dataset, only $D_{R}=20$ is used as explained before. The results are shown for decreasing values of SNR expressed in dB. The results presented in this subsection are calculated using the Decision Tree algorithm for the 1 K dataset and the Support Vector Machine algorithm for the PNE and GUNSHOT datasets. The justification for such choice is provided in Section 5.3.

**Figure 6:**
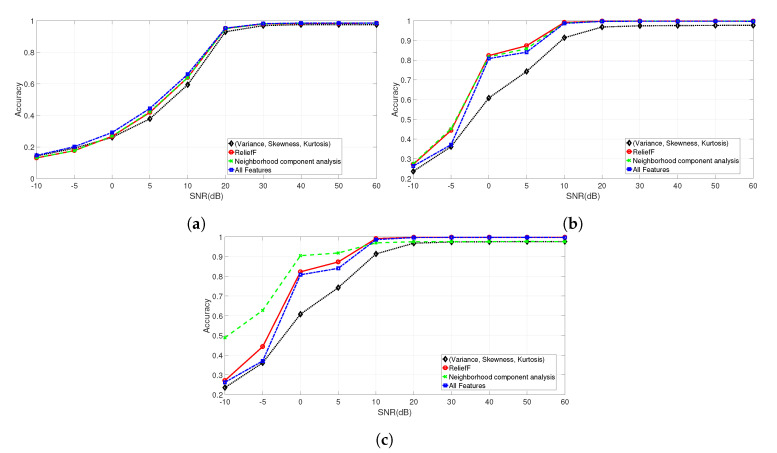
Comparison of the accuracy among different sets of features for the 1 K, PNE, and GUNSHOT datasets for decreasing values of Signal to Noise Ratio (SNR) (expressed in dB). (**a**) Accuracies for the 1 K dataset with $D_{R}=20$ with different set of features; (**b**) Accuracies for the PNE dataset with $D_{R}=20$ with different set of features; (**c**) Accuracies for the GUNSHOT dataset with $D_{R}=20$ with different set of features.

Figure 6a–c show the accuracy for the 1 K, PNE, and GUNSHOT datasets with $D_{R}=20$ using the four different sets of features.

**Figure 7:**
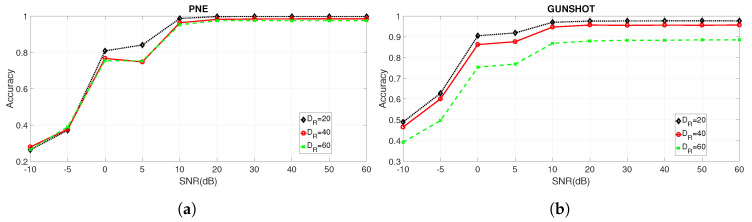
Comparison of the accuracy obtained for the PNE and GUNSHOT datasets for decreasing values of SNR expressed in dB and different values of DR. (**a**) PNE dataset with $D_{R}=20$, $D_{R}=40$ and $D_{R}=60$; (**b**) GUNSHOT dataset with $D_{R}=20$, $D_{R}=40$ and $D_{R}=60$.

Figure 7a,b show, respectively, the accuracy for the PNE and GUNSHOT datasets for different values of $D_{R}$: $D_{R}=20$,$D_{R}=40$ and $D_{R}=60$.

**Figure 8:**
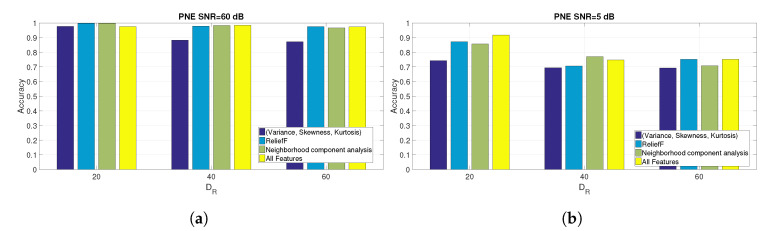
Comparison of the accuracy obtained for the PNE dataset at SNR = 60 dB and SNR = 5 dB for for different set of features and for different values of $D_{R}$. (**a**) PNE dataset at SNR = 60 dB; (**b**) PNE dataset at SNR = 5 dB.

**Figure 9:**
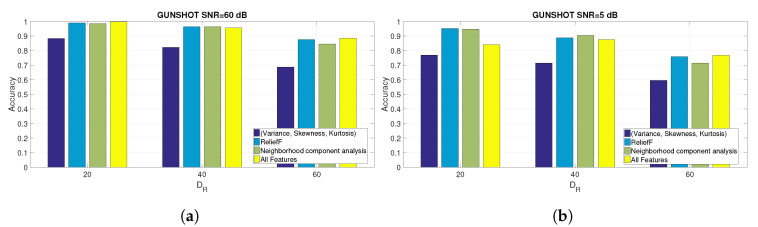
Comparison of the accuracy obtained for the GUNSHOT dataset at SNR = 60 dB and SNR = 5 dB for different set of features and for different values of $D_{R}$. (**a**) Comparison of the accuracy for the GUNSHOT dataset at SNR = 60 dB for different set of features and for different values of $D_{R}$; (**b**) Comparison of the accuracy for the GUNSHOT dataset at SNR = 5 dB for different set of features and for different values of $D_{R}$.

Because the values reported in Figure 7a,b are quite close (at least for Figure 7a), additional figures are presented (Figure 8a,b for the PNE dataset and Figure 9a,b for the GUNSHOT dataset) to show the accuracy for specific values of SNR expressed in dB and for the different sets of features.

The results evidenced from the Figures and the Table show that the combination of the identified features is able to obtain a very high identification accuracy (near 100%) in optimal SNR conditions (e.g., SNR = 60 dB). On the other side, the parameter $D_{R}$ has a significant impact on the identification accuracy in all datasets because the accuracy degrades significantly for higher values of $D_{R}$. The reason is that the machine learning algorithm has to operate on a smaller set of data (e.g., the entropy measures extracted from the spectral representation of the audio recordings). On the other side, the classification time (as shown later in Section 5.3) is inversely proportional to $D_{R}$. Then, a trade-off is present between classification accuracy and classification time.

Other considerations based on the analysis of the results presented in Table 7, Figure 6a–c and Figure 7 are presented in the following bullet list:

- The selected features obtained from the ReliefF and NCA algorithms provide a significant improvement in classification performance respect to the basis statistical features (variance, skewness, and kurtosis) and to the overall set of features (for the PNE and GUNSHOT datasets) for SNR ≤20 dB. The improvement is minor but still significant for the case of the 1 K dataset (see Figure 6a). For values of SNR >20 dB, the accuracy of all the methods tend to converge to very high values (i.e., accuracy ≥0.98) as shown in the summary Table 7. From one side, this result shows that an entropy based approach is able to obtain in general (e.g., with different sets of entropy features) a very high classification accuracy even with a significant dimensionality reduction. It performs well in comparison with more sophisticated deep learning methods (see Reference [18]), which require significant computational power. On the other side, robustness to noise is a very desirable feature in practical applications of the identification technique presented in this paper because of the presence of background noise or distance between the generator of the audio stimulus and the microphone. From this point of view, a careful selection of features improve significantly the robustness of the proposed method. Further details are provided in the paragraphs below.
- We note the discriminating power of the entropy measures against the variance, skewness, and kurtosis features, which proves one of the assumptions of the study that entropy measures have an high discriminant power, and they can be effectively used for the classification of microphones. The advantage of the use of entropy measures is particularly important for the PNE and GUNSHOT dataset (Figure 6b,c), and it is less significant for the 1 K dataset (Figure 6a). This may due to the limited frequency range of the 1 K stimulus as shown in Figure 1b.
- The accuracy obtained for the 1 K dataset is lower than the accuracy obtained for the PNE and GUNSHOT datasets as shown in Table 7. This may due to the more extensive frequency response of the microphones when they are stimulated by the PNE and GUNSHOT stimuli in comparison to the single tone at 1 KHz. In addition, the robustness of the classification algorithm against AWGN is also minor in comparison to the PNE and GUNSHOT datasets for similar reasons.
- Table 7 shows that the application of entropy measures is particularly robust in the presence of Gaussian noise for the PNE and the GUNSHOT datasets. Even at SNR = 0 dB, the accuracy is still more than 90% for the GUNSHOT dataset using the NCA algorithm and more than 80% for the PNE dataset. For the 1 K dataset, the classification algorithm is not particularly robust: around 30% for SNR = 0 dB.
- The performance of the ReliefF and NCA algorithms is quite similar for the 1 K and PNE datasets (Figure 6a,b, while the NCA algorithm is significantly better (from the point of view of robustness to noise) than the ReliefF algorithm for the GUNSHOT dataset (Figure 6c).
- An analysis of Table 4 and Table 5 (which identify the entropy features used in the analysis) with Table 7, which reports the accuracy for different values of $D_{R}$ and SNR shows that Shannon Entropy and Renyi Entropy are important features with lower downsampling rate ($D_{R}=20$), while Shannon Entropy, Dispersion Entropy, and Permutation Entropy correspond to important features in the other downsampling rates ($D_{R}=40$ and $D_{R}=60$). A potential explanation of this result is that the Renyi entropy is robust for time series with strong nonlinearity and nonstationarity as in this case of a spectral representation of the audio recordings. In addition, Renyi Entropy is a generalization (for different values of α from Equation (3)) of the classical Shannon Entropy, which is also shown from the results in Table 7 to perform well. The strong performance of the Dispersion Entropy and Permutation Entropy can be explained by the consideration that the frequency response is correlated with the electronic components in the microphones, whose specific characteristics (mapped to the fingerprints) may appear in frequency patterns. For example, an amplifier in the model of a microphone can have a specific pattern of frequencies, which is present in all the microphones of that model and which can be used as a fingerprint. Since the Dispersion Entropy and Permutation Entropy are based on the frequency of patterns in the time series, they can capture this specific information in a more effective way than other entropy measures. In addition, Permutation Entropy and Dispersion Entropy are also particularly robust to the presence of noise [19,37].

### 5.3. Evaluation of Machine Learning Algorithms

The previous results (Figure 6a–c and Table 7) were obtained using the Decision Tree machine learning algorithm for the 1 K dataset and the Support Vector Machine algorithm for the PNE and GUNSHOT datasets. This choice is justified by the assessment of the different machine learning algorithms presented in this subsection.

**Figure 10:**
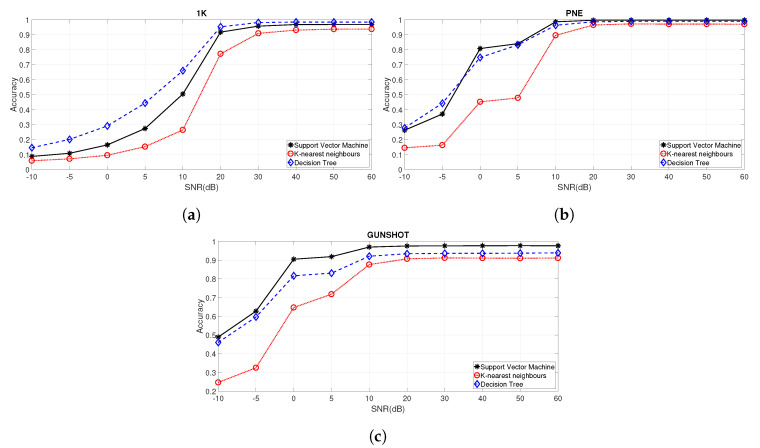
Comparison of the three machine learning algorithms for the three considered datasets. (**a**) Comparison among SVM, KNN, and DT using the 1 K dataset with all features for $D_{R}=20$. The accuracy is reported; (**b**) Comparison of the accuracy among SVM, KNN, and DT using the PNE dataset with all features for $D_{R}=20$; (**c**) Comparison of the accuracy among SVM, KNN, and DT using the GUNSHOT dataset with all features for $D_{R}=20$.

A comparison of the results for the three different machine learning algorithms identified in Section 3.4 are shown in Figure 10a–c, respectively, for the 1 K, PNE, and the GUNSHOT datasets for different values of SNR.

It can be seen that SVM outperforms both KNN and Decision Tree (DT) across most of the values of SNR for the PNE and the GUNSHOT datasets. For the 1 K dataset the Decision Tree outperforms the SVM and KNN. The values of the hyperparameters of the algorithms and the range on which they were optimized is reported in Table 2.

**Figure 11:**
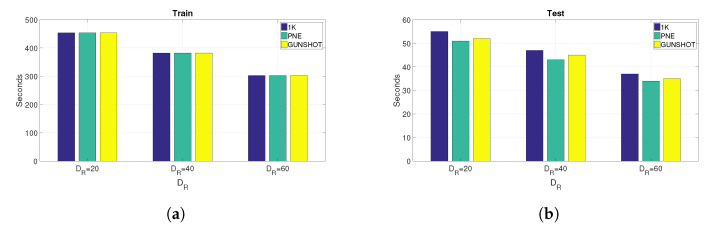
Computing time (application of entropy measures plus classification time) for all datasets and values of $D_{R}$ using the Support Vector Machine algorithm and all features. (**a**) Application of entropy measures plus training time, (**b**) Application of entropy measures plus test time.

The time needed to calculate the entropy measures and to perform the classification process (training and testing) is shown for different values of $D_{R}$, and it is reported, respectively, for training in Figure 11a and test in Figure 11b. The computing time was based on the application of the Support Vector Machine algorithm and with all the features. The computing platform used to calculate the estimated processing time in seconds is a laptop computer with an Intel Core i7 9700 K running at a clock speed of 3.6 GHz with 32 GBytes of RAM.

### 5.4. Confusion Matrices Analysis

**Figure 12:**
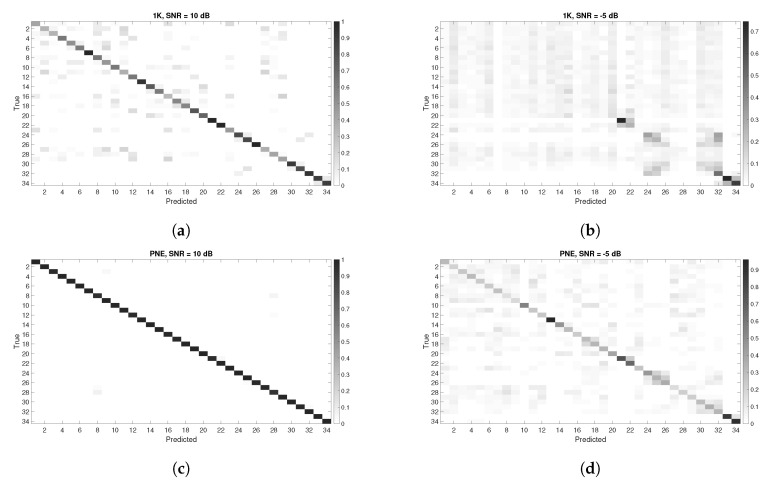

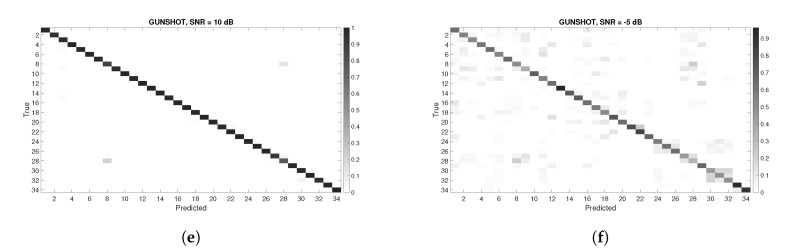
Confusion Matrices for the three datasets using **all features**. Y axis represents the instances of the True classes, while the X axis represents the instances of the Predicted classes. (**a**) All features scenario. Confusion Matrix with $D_{R}=20$ and SNR = 10 dB, 1 K dataset with all features; (**b**) Confusion Matrix with $D_{R}=20$ and SNR = −5 dB, 1 K dataset with all features; (**c**) Confusion Matrix with $D_{R}=20$ and SNR = 10 dB, PNE dataset with all features; (**d**) Confusion Matrix with $D_{R}=20$ and SNR = −5 dB, PNE dataset with all features; (**e**) Confusion Matrix with $D_{R}=20$ and SNR = 10 dB, GUNSHOT dataset with all features; (**f**) Confusion Matrix with $D_{R}=20$ and SNR = −5 dB, GUNSHOT dataset with all features.

**Figure 13:**
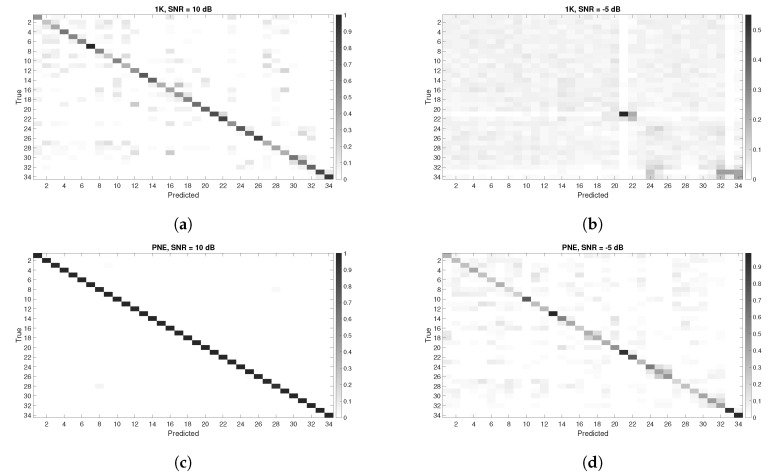

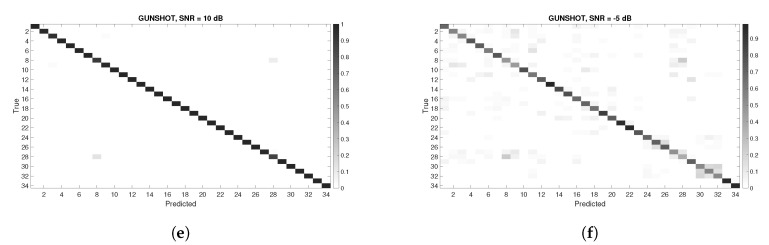
Confusion Matrices for the three datasets with the **best features using the ReliefF approach**. Y axis represents the instances of the True classes, while the X axis represents the instances of the Predicted classes. (**a**) Confusion Matrix with $D_{R}=20$ and SNR = 10 dB, 1 K dataset; (**b**) Confusion Matrix with $D_{R}=20$ and SNR = −5 dB, 1 K dataset; (**c**) Confusion Matrix with $D_{R}=20$ and SNR = 10 dB, PNE dataset; (**d**) Confusion Matrix with $D_{R}=20$ and SNR = 5 dB, PNE dataset; (**e**) Confusion Matrix with $D_{R}=20$ and SNR = 10 dB, GUNSHOT; (**f**) Confusion Matrix with $D_{R}=20$ and SNR = 5 dB, GUNSHOT.

Classification accuracy is a widely used as benchmark but it does not provide the complete assessment of the machine learning algorithm performance. Then, in this section, we provides the confusion matrices on some of the previous results. We provide in the Figure 12 and Figure 13, the confusion matrices for different values of $D_{R}$ and various SNR values (expressed in dB) for all the datasets. Lower values of SNR were selected because they highlight in a more descriptive way the number of False Positives (FP)s and False Negatives (FN)s. For very high values of SNR (e.g., SNR = 50 dB), the classification accuracy is so high that the confusion matrix does not provide useful information.

The results presented in the confusion matrices hereafter show that the number of FPs and FNs increases with lower values of SNR but also with $D_{R}=40$.

The following conclusions can be drawn from Figure 12 and Figure 13:

- We do not notice any specific asymmetry (higher values of FP against FN, or vice versa), both for the PNE and GUNSHOT datasets, which also points out that the accuracy metric has some degree of reliability.
- Intra-model classification has a lower accuracy than inter-model accuracy. For example, Figure 12b shows that the first 23 phones (Id from 1 to 23) of the same model are very difficult to distinguish among themselves. In a similar way, the triplet phones of other models (e.g., HTC One X, Samsung Galaxy, Sony Experia) are also difficult to distinguish among themselves (they are easy to be visually identified in the confusion matrix as squares). One exception is the Google Nexus, which can be identified with high accuracy from the other phones and among themselves even in low SNR conditions. A technical analysis of the Google Nexus has shown a significant different design from the other phones for the microphone component, which may explain the findings.
- The intra-model classification of the Samsung ACE is more difficult to achieve in the PNE dataset rather than the GUNSHOT dataset. This is visible by comparing Figure 13f with Figure 13d. Even if the number of FPs and FNs is quite high, the Samsung ACE phones can be more easily distinguished in the GUNSHOT data (darker color in the diagonal of the confusion matrix for the first 23 phones). One potential reason for this is that the GUNSHOT audio stimulus has a wider extension in the spectral domain than the PNE dataset, and the Samsung ACE phones have a frequency response which can be exploited by the classification algorithm and from the entropy measures in a different way.

### 5.5. Receiver Operating Characteristics

While the focus of this paper is on the identification problem to identify a microphone, among others, this section provides a brief analysis on the capability of the proposed approach to distinguish a microphone from another. In particular, we identify two classification scenarios. The first is an intra-model scenario where two microphones from mobile phones of the same model (Phone 1 and Phone 2 of the model Samsung ACE) are compared. The second is an inter-model scenario where the two microphones are from mobile phones of different models (Phone 1 of model Samsung ACE and Phone 24 of model HTC One X). For these two scenarios, we estimated the Receiver Operative Characteristics (ROC)s with $D_{R}=20$ and across different values of SNR expressed in dB. The best set of features from Table 6 using NCA are used to calculate the ROCs. The SVM algorithm is used for the binary classification. The ROC curve is a graphical plot that describes the discriminating capability of a binary classifier system as its discrimination threshold is varied. The ROC curve is created by plotting the true positive rate (TPR) against the false positive rate (FPR) at various threshold settings. The more the ROC curve is aligned towards the diagonal between FPR/TPR = 0 and FPR/TPR = 1 and the worst is the classification performance. Apart from a qualitative analysis of the ROC curves (e.g., looking at the ROC shape), a quantitative metric to assess the classification performance is the Equal Error Rate (EER), which is the point where FPR = 1 − TPR, and it can be measured with the corresponding value of the FPR, as in this paper.

**Figure 14:**
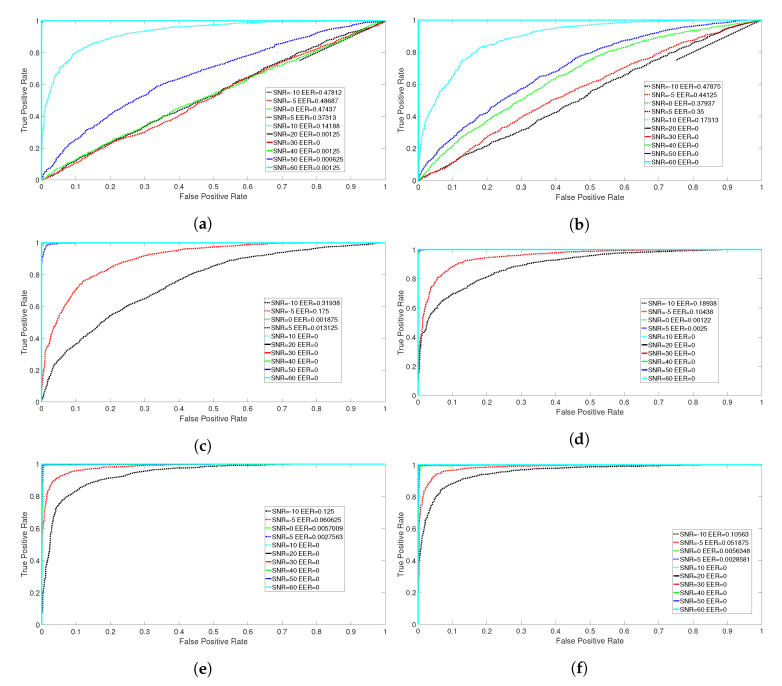
Receiver Operative Characteristics (ROCs) between different sets of microphones with $D_{R}=20$ and different datasets. Selected features using NCA. SVM is used for binary classification. (**a**) ROC between microphone 1 and 2 (Samsung Ace). 1 K dataset; (**b**) ROC between microphone 1 and 24 (Samsung Ace and HTC ONE X). 1 K dataset; (**c**) ROC between microphone 1 and 2 (Samsung Ace). PNE dataset; (**d**) ROC between microphone 1 and 24 (Samsung Ace and HTC ONE X). PNE dataset; (**e**) ROC between microphone 1 and 2 (Samsung Ace and HTC ONE X). GUNSHOT dataset; (**f**) ROC between microphone 1 and 24 (Samsung Ace and HTC ONE X). GUNSHOT dataset.

The results for the two scenarios are presented in Figure 14 and, more specifically, in Figure 14a,c,e, respectively, for the datasets 1 K, PNE, and GUN for the intramodel scenario (phone 1 and phone 2), while the inter-model scenario (phone 1 and phone 24) is shown in Figure 14b,d,f, respectively, for the datasets 1 K, PNE, and GUN.

The results presented in Figure 14 confirm the previous accuracy results: for lower values of SNR expressed in dB, the method is not able to distinguish accurately the two microphones. In particular, the presence of noise is particularly significant for the 1 K dataset (as also anticipated by the results for accuracy presented in Figure 6a) as shown in Figure 14a,b. All the sub-figures in Figure 14 show the corresponding values of EER in the legend. It can be seen that for high values of SNR in dB, the approach proposed in this paper manages to achieve full distinction of the microphones (EER = 0). For this reason, the ROC curves for high values of SNR in dB overlap, and not all the ROC curves (for different values of SNR) are visible in the sub-figures of Figure 14.

By comparing the Figures for the intra-model scenario (Figure 14a,c,e) and the inter-model scenario (Figure 14b,d,f), it can be seen that the approach has more difficulty to distinguish microphones belonging to the same model rather than different models. This can be seen by the values of EER in the legends. The values of EER are generally higher for intra-model classification in comparison to the inter-model classification for different values of the SNR in dB. For example, EER is equal to 0.175 for SNR = −5 dB in Figure 14c (i.e., PNE intramodel), while EER is equal to 0.10438 in Figure 14d (i.e., PNE intermodel). This is not surprising and it is confirmed by the results in literature [11,16,18]. It is due to the reason that microphones belonging to two different models have different design, materials and manufacturing processes which (in turn) generate significantly different fingerprints, which is not the case for microphones belonging to the same model. A more detailed analysis on the binary classification among different microphones will be pursued in future developments.

## 6. Conclusions and Future Developments

This paper evaluated the applicability of entropy measures to the problem of identification of microphones, which are built-in in mass market mobile phones. The entropy measures are extracted from the spectral representation (complex magnitude) of the audio recordings of the phones stimulated with three different types of sounds. The results show that entropy measures together with statistical features can provide a very high identification accuracy with the advantage that the feature extraction process implements a dimensionality reduction with decreased classification time. An analysis on the impact of downsampling and the hyperparameters of the entropy measures was performed to identify the most discriminating elements and the entropy measures with highest discriminating power. In particular, the results show that Shannon Entropy and Renyi Entropy are important features with lower downsampling rate ($D_{R}=20$), while Shannon Entropy, Dispersion Entropy, and Permutation Entropy correspond to important features in the higher downsampling rates ($D_{R}=40$ and $D_{R}=60$). The analysis done in this work can be extended in the future to different types of sound stimuli, to other types of entropy measures, or other types of electronic devices.

Future developments will investigate the application of time-frequency analysis and transforms (e.g., spectrograms) in combination to entropy measures. In particular, the impact of the hyper-parameters in the definition of the time frequency transforms will be evaluated. Because the application of time-frequency transform enlarges the size of the data to be processed, additional dimensionality reduction techniques or efficient feature selection techniques will be investigated to maintain the overall processing time feasible for practical applications. In addition, future studies will investigate more in detail the authentication problem to mitigate a spoofing attack where a rogue microphone is used to emulated a legitimate microphone. In this context, we will also investigate how the entropy measures can be used to generate secret keys in a similar way to the Physical Unclonable Function (PUF) concept.

## Figures and Tables

**Table 1:** List of the 34 mobile phones used in the experiments with relative IDentifiers (IDs).

Mobile Phones	IDs	Quantity
Samsung ACE	from 1 to 23	23
HTC One X	from 24 to 26	3
Samsung Galaxy S5	from 27 to 29	3
Sony Experia	from 30 to 32	3
Google Nexus	from 33 to 34	2
**Total**		**34**

**Table 2:** Hyperparameters of the machine learning algorithms.

Machine Learning Algorithm	First Hyperparameter	Second Hyperparameter
**1 K dataset**		
SVM	RBF scaling factor $\gamma =2^{4}$ (range: $2^{1}$ to $2^{10}$)	C factor = $2^{7}$ (range: $2^{1}$ to $2^{10}$)
KNN	K = 2 (range: 1 to 10)	Distance metric = Euclidean Distance (choices: Chebychev, Euclidean, Minkowski)
Decision Tree	Maximum Number of splits = 10 (range: 2 to 12)	Split Criterion = Gini’s diversity index (choices: Gini Index and Cross-Entropy)
**PNE dataset**		
SVM	RBF scaling factor $\gamma =2^{5}$ (range: $2^{1}$ to $2^{10}$)	C factor = $2^{6}$ (range: $2^{1}$ to $2^{10}$)
KNN	K = 1 (range: 1 to 10)	Distance metric = Euclidean Distance (choices: Chebychev, Euclidean, Minkowski)
Decision Tree	Maximum Number of splits = 8 (range: 2 to 12)	Split Criterion = Gini’s diversity index (choices: Gini Index and Cross-Entropy)
**GUNSHOT dataset**		
SVM	RBF scaling factor $\gamma =2^{5}$ (range: $2^{1}$ to $2^{10}$)	C factor = $2^{9}$ (range: $2^{1}$ to $2^{10}$)
KNN	K = 1 (range: 1 to 10)	Distance metric = Euclidean Distance (choices: Chebychev, Euclidean, Minkowski)
Decision Tree	Maximum Number of splits = 8 (range: 2 to 12)	Split Criterion = Gini’s diversity index (choices: Gini Index and Cross-Entropy)

**Table 3:** Combinations of *m* and *c* used for each value of $D_{R}$ to define the applied entropy measures.

$D_{R}$	Parameters Combination
$D_{R}=20$	(m=2,c=3), (m=2,c=4), (m=3,c=3), (m=3,c=4)
$D_{R}=40$	(m=2,c=3), (m=3,c=3)
$D_{R}=60$	(m=2,c=3), (m=3,c=3)

**Table 4:** List of features used in the analysis with $D_{R}=20$.

Feature Id	Description of the Feature	Hyperparameter
1	Standard Deviation	None
2	Skewness	None
3	Kurtosis	None
4	Renyi	2
5	Renyi	3
6	Renyi	4
7	Shannon Entropy	No hyperparameter
8, 24, 25, 41	Dispersion Entropy	(m=2,c=3) and (m=2,c=4) and (m=3,c=3) and (m=3,c=4)
9, 26	Permutation Entropy	(m=2) and (m=3)
10 and 27	Approximate Entropy	(m=2) and (m=3), *r* = 0.03 Standard Deviation
11, 28	Approximate Entropy	(m=2) and (m=3), *r* = 0.05 Standard Deviation
12, 29	Approximate Entropy	(m=2) and (m=3), *r* = 0.1 Standard Deviation
13, 30	Approximate Entropy	(m=2) and (m=3), *r* = 0.2 Standard Deviation
14, 31	Approximate Entropy	(m=2) and (m=3), *r* = 0.3 Standard Deviation
15, 32	Approximate Entropy	(m=2) and (m=3), *r* = 0.4 Standard Deviation
16, 33	Sample Entropy	(m=2) and (m=3), *r* = 0.1 Standard Deviation
17, 34	Sample Entropy	(m=2) and (m=3), *r* = 0.2 Standard Deviation
18, 35	Sample Entropy	(m=2) and (m=3), *r* = 0.1 Standard Deviation
19, 36	Sample Entropy	(m=2) and (m=3), *r* = 0.2 Standard Deviation
20, 37	Fuzzy Entropy	(m=2) and (m=3), *r* = 0.1 Standard Deviation
21, 38	Fuzzy Entropy	(m=2) and (m=3), *r* = 0.2 Standard Deviation
22, 39	Fuzzy Entropy	(m=2) and (m=3), *r* = 0.1 Standard Deviation
23, 40	Fuzzy Entropy	(m=2) and (m=3), *r* = 0.2 Standard Deviation

**Table 5:** List of features used in the analysis with $D_{R}=40$ and $D_{R}=60$.

Feature Id	Description of the Feature	Hyperparameter
1	Standard Deviation	None
2	Skewness	None
3	Kurtosis	None
4	Renyi	2
5	Renyi	3
6	Renyi	4
7	Shannon Entropy	No hyperparameter
8, 24	Dispersion Entropy	(m=2,c=3) and (m=3,c=3)
9, 25	Permutation Entropy	(m=2 and m=3)
10, 26	Approximate Entropy	(m=2 and m=3), *r* = 0.03 Standard Deviation
11, 27	Approximate Entropy	(m=2 and m=3), *r* = 0.05 Standard Deviation
12, 28	Approximate Entropy	(m=2 and m=3), *r* = 0.1 Standard Deviation
13, 29	Approximate Entropy	(m=2 and m=3), *r* = 0.2 Standard Deviation
14, 30	Approximate Entropy	(m=2 and m=3), *r* = 0.3 Standard Deviation
15, 31	Approximate Entropy	(m=2 and m=3), *r* = 0.4 Standard Deviation
16, 32	Sample Entropy	(m=2 and m=3), *r*=0.1 Standard Deviation
17, 33	Sample Entropy	(m=2 and m=3), *r* = 0.2 Standard Deviation
18, 34	Sample Entropy	(m=2 and m=3), *r* = 0.1 Standard Deviation
19, 35	Sample Entropy	(m=2 and m=3), *r* = 0.2 Standard Deviation
20, 36	Fuzzy Entropy	(m=2 and m=3), *r* = 0.1 Standard Deviation
21, 37	Fuzzy Entropy	(m=2 and m=3), *r* = 0.2 Standard Deviation
22, 38	Fuzzy Entropy	(m=2 and m=3), *r* = 0.1 Standard Deviation
23, 39	Fuzzy Entropy	(m=2 and m=3), *r* = 0.2 Standard Deviation

**Table 6:** Optimal sets of features for the different datasets and different values of $D_{R}$.

Data Set, $D_{R}$	Method	Optimal Features Set
$D_{R}$ **= 20**		
1 K	ReliefF	[4,5,6,7,12,13,14,15,31,32]
PNE	ReliefF	[1,2,3,5,6,7,8,13,27,29]
GUNSHOT	ReliefF	[1,2,4,5,6,7,12,24,26,29]
1 K	NCA	[1,4,5,6,7,12,13,14,15,31,32]
PNE	NCA	[1,2,3,5,6,7,8,14,15,32]
GUNSHOT	NCA	[1,2,4,5,6,7,11,21,22,23]
$D_{R}$ **= 40**		
PNE	ReliefF	[1,2,5,7,8,9,16,17,19,35]
GUNSHOT	ReliefF	[1,2,5,6,7,8,14,16,35,37]
PNE	NCA	[1,2,5,6,7,8,9,14,30,32]
GUNSHOT	NCA	[1,4,5,6,7,12,13,14,29,30]
$D_{R}$ **= 60**		
PNE	ReliefF	[1,2,3,4,6,7,8,9,18,37]
GUNSHOT	ReliefF	[1,2,3,4,6,7,8,16,18,37]
PNE	NCA	[1,2,6,7,8,9,17,18,37,38]
GUNSHOT	NCA	[1,5,6,7,9,16,18,19,38,39]

**Table 7:** Accuracy results for different values (in dB) of the SNR for different set of features. The highest values are highlighted in bold.

Dataset and SNR Value (dB)	All Features	Selected Features with NCA (Best 10 Features)	Selected Features with ReliefF (Best 10 Features)	Baseline Statistical Features
$D_{R}=20$				
1 K SNR=−5 dB	**0.2015**	0.1763	0.1768	0.1917
1 K SNR=10 dB	**0.6602**	0.6399	0.6396	0.5943
1 K SNR=30 dB	**0.9816**	0.9799	0.9791	0.9688
1 K SNR=60 dB	0.9846	**0.9851**	0.9848	0.9748
PNE SNR=−5 dB	0.371	**0.4521**	0.4436	0.3615
PNE SNR=10 dB	0.9863	0.9883	**0.9915**	0.9137
PNE SNR=30 dB	0.9971	0.9978	**0.9982**	0.9741
PNE SNR=60 dB	0.9974	0.9978	**0.9982**	0.9764
GUNSHOT SNR=−5 dB	0.6274	0.6943	**0.7039**	0.5240
GUNSHOT SNR=10 dB	0.9696	0.9816	**0.9857**	0.8631
GUNSHOT SNR=30 dB	0.9761	0.9851	**0.9889**	0.8800
GUNSHOT SNR=60 dB	0.9762	0.9857	**0.9890**	0.8835
$D_{R}=40$				
PNE SNR=−5 dB	0.3760	**0.4069**	0.3621	0.3818
PNE SNR=10 dB	**0.9629**	0.9616	0.9567	0.8409
PNE SNR=30 dB	**0.9834**	0.9815	0.9793	0.8826
PNE SNR=60 dB	0.9840	**0.9856**	0.9784	0.8821
GUNSHOT SNR=−5	0.6004	**0.6587**	0.6238	0.509
GUNSHOT SNR=10	0.9465	**0.9558**	0.9531	0.8071
GUNSHOT SNR=30 dB	0.9549	**0.9647**	0.9632	0.8291
GUNSHOT SNR=60 dB	0.9565	**0.9648**	0.9637	0.8228
$D_{R}=60$				
PNE SNR=−5 dB	0.3890	0.3818	**0.4078**	0.3842
PNE SNR=10 dB	0.9520	0.9397	**0.9532**	0.8302
PNE SNR=30 dB	0.9750	0.9655	**0.9751**	0.8745
PNE SNR=60 dB	0.9746	0.9668	**0.9756**	0.8719
GUNSHOT SNR=−5	0.4963	0.4731	**0.5110**	0.4332
GUNSHOT SNR=10 dB	**0.8682**	0.8190	0.8535	0.6780
GUNSHOT SNR=30 dB	**0.8829**	0.8466	0.8714	0.6940
GUNSHOT SNR=60 dB	**0.8851**	0.8451	0.8755	0.6875

## Abbreviations

The following abbreviations are used in this manuscript:

AWGN	Additive White Gaussian Noise
CNN	Convolutional Neural Networks
DiEn	Dispersion Entropy
DT	Decision Tree
ECOC	Error-Correcting Output Codes
EER	Equal Error Rate
FP	False Positives
FN	False Negatives
PeEn	Permutation Entropy
KNN	K Nearest Neighbor
LOO	Leave-One-Out
LOOCV	Leave-One-Out Cross-Validation
MFCC	Mel-Frequency Cepstrum Coefficient
NCA	Neighborhood Component Analysis
NCDF	Normal Cumulative Distribution Function
PCM	Pulse Code Modulation
PUF	Physical Unclonable Function
RAM	Random Access Memory
RBF	Radial Basis Function
ROC	Receiver operating characteristic
SaEn	Sample Entropy
ShEn	Shannon Entropy
SEI	Special Emitter Identification
SNR	Signal Noise Ratio
SVM	Support Vector Machine
